# Exploring anterion capsular contraction syndrome in cataract surgery: insights into pathogenesis, clinical course, influencing factors, and intervention approaches

**DOI:** 10.3389/fmed.2024.1366576

**Published:** 2024-02-19

**Authors:** Xuanqiao Lin, Dongmei Ma, Jin Yang

**Affiliations:** ^1^Eye Institute and Department of Ophthalmology, Eye & ENT Hospital, Fudan University, Shanghai, China; ^2^Key NHC Laboratory of Myopia, Fudan University, Laboratory of Myopia, Chinese Academy of Medical Sciences, Shanghai, China; ^3^Shanghai Key Laboratory of Visual Impairment and Restoration, Shanghai, China; ^4^Eye Hospital and School of Ophthalmology and Optometry, Wenzhou Medical University, Wenzhou, China

**Keywords:** capsular contraction syndrome, cataract surgery, intraocular lenses, capsular tension ring, laser anterior capsulotomy

## Abstract

Anterior capsular contraction syndrome (ACCS) is a challenging complication that can occur following phacoemulsification cataract surgery. Characterized by capsular bag wrinkling, intraocular lens (IOL) decentration and tilt, ACCS can have negative effects on visual outcomes and patient satisfaction. This review aims to investigate the pathogenesis, clinical course, influencing factors, and intervention approaches for ACCS after cataract surgery. By understanding the underlying mechanisms and identifying factors that contribute to ACCS, surgeons can enhance their ability to predict and manage this complication. Various intervention strategies are discussed, highlighting their importance in reducing complications and improving surgical outcomes. However, further research is needed to determine optimal prevention and management strategies through long-term follow-up and comparative analyses. Advancements in this field will ultimately lead to improved visual outcomes and optimized cataract surgery for patients.

## 1 Introduction

In modern cataract surgery techniques, phacoemulsification has become the preferred method for lens extraction (1, 2). However, as these techniques have been widely applied, it has been increasingly recognized that there are some less common postoperative complications (3). Anterior capsular contraction syndrome (ACCS), characterized by capsular wrinkling, fibrosis leading to reduced equatorial diameter of the capsular bag, intraocular lens (IOL) decentration and tilt, was initially named by Davison (4). The ACCS will have a negative impact on the patient’s vision and visual quality. In severe cases, it may also lead to IOL dislocation (5–7). As a result, research on the factors influencing ACCS and intervention measures has become the foundation for ensuring postoperative visual quality stability in patients, especially for high myopia, retinitis pigmentosa (RP), and other high-risk patients. Therefore, this article provides a comprehensive review of the research progress, influencing factors, and intervention measures for ACCS following cataract surgery (Table 1).

**Table 1:** Influencing factors for ACCS in this review.

	Risk factors	Prevention factors
CCC factor	Oversized or undersized capsulorhexis area diameter	Suitable capsulorhexis area diameter
IOL factors	Hydrophilic material	Hydrophobic material
	Single-piece design	“Peak-like” shape edge design
	C-Loop haptic	Plate haptic
	Hinge-based accommodative IOL	More haptics IOL
Diseases factors	High myopia	No Relevant Diseases
	RP	
	Uveitis	
	PEX syndrome	
	Diabetes	
	Dystrophia myotonica	
Intervention factors	No Intervention Conducted	Anterior capsule polishing
		Implantation of CTR
		IOL modification
		Anti-inflammatory medications
		Prophylactic laser anterior capsulotomy
Other factors	Advanced age	

ACCS, anterior capsular contraction syndrome; CCC, continuous curvilinear capsulorhexis; IOL, intraocular lens; RP, retinitis pigmentosa; PEX, pseudoexfoliation; CTR, capsular tension ring.

## 2 Pathogenesis of ACCS

The pathogenesis of ACCS is not fully understood but can be primarily categorized into two aspects: cellular and mechanical.

The cellular pathogenesis involves various factors such as trauma during cataract surgery, inflammatory response following IOL implantation, disruption of the blood-aqueous barrier and blood-retinal barrier, and stimulation from IOLs. These factors can stimulate residual lens epithelial cells (LECs) present at the capsulorhexis opening, leading to the production of excessive ECM, mainly collagen fibers (8). The proliferation and transdifferentiation of LECs ultimately result in ACCS, posterior capsule opacity (PCO), and other complications. In this process, the involvement of various cytokines has been confirmed, including interleukin (IL), transforming growth factor-beta (TGF-β), and alpha-smooth muscle actin (α-SMA) (9, 10). For instance, the inability of TGF-β2 in the aqueous humor to inhibit LECs activity and induce apoptosis due to the obstruction of aqueous humor circulation caused by IOL implantation leads to LECs proliferation (11, 12).

As for mechanical studies, it has been indicated that the decrease or enlargement of the capsulorhexis area is associated with the resultant centrifugal force from the contraction of normal lens fibers and the resultant centripetal force from the contraction of lens fibers at the edge of the capsulorhexis area (13, 14). Therefore, ACCS occurs in patients with weaken zonules because of the imbalance between the centrifugal force generated by the contraction of zonules and the centripetal force generated by the contraction of lens fibers at the edge of the capsulorhexis area. This supports the application of capsular tension ring (CTR) for intervening ACCS (14, 15). Wang et al. (16) suggested that the mechanism by which Neodymium:YAG (Nd:YAG) anterior capsulotomy prevents further deterioration of ACCS may be related to the relaxation of stress on the anterior capsule following continuous curvilinear capsulorhexis (CCC). The biomechanical mechanisms of ACCS need to be given sufficient attention in future researches.

## 3 Incidence and course of ACCS

Early studies have shown that the incidence of ACCS ranges from 1.4% to 14.0%, with most cases occurring within 3 to 30 weeks after surgery. After 3 months, the contraction of the capsular bag slows down and tends to stabilize (4, 17). However, with recent in-depth studies, we have found that the course of ACCS can develop in the weeks to years following surgery and in high-risk patients such as those with pseudoexfoliation (PEX), high myopia, and RP, the incidence of ACCS may increase to 10% to 30% or more (18, 19). Furthermore, a comprehensive population study with a 30 years follow-up indicates that late in-the-bag IOL dislocation can occur after cataract surgery, ranging from 6 months to 25 years or even longer, and it is associated with zonular dehiscence and capsular bag contraction (20, 21).

However, not all cases of ACCS will necessarily result in IOL dislocation. The progression of ACCS can be divided into multiple stages (4, 7). In the early stage (approximately less than 1 month) of ACCS, the patient exhibits fibrosis of the capsulorhexis edge, along with mild thickening and opacification of the anterior capsule. Wrinkling of the capsular bag is observed, resulting in a slight reduction in the area of CCC. Visual acuity and contrast sensitivity remain unaffected, and the patient does not experience significant glare symptoms. With the continuous progression of capsular bag contraction, the area of CCC significantly decreases. The degree of contraction varies in different quadrants, with the opening tending to shrink more rapidly in the corresponding quadrants. This leads to a slight displacement of IOL. At this point, due to the change in the position of IOL, there is a change in refractive status. Research has shown that when the decentration of the IOL exceeds 1.0 mm or the tilt angle is greater than 5 degrees, it will have an impact on vision (22, 23). Additionally, the contraction of the capsule causes wrinkling of the posterior capsule, resulting in the Maddox rod effect, which can induce glare. As time passes, the area of the anterior capsular opening continues to decrease due to the contraction of the capsular bag, leading to compression on the haptics, causing them to bend and resulting in more significant eccentricity and displacement (24). Finally, when the zonules are unable to withstand the contraction of the capsular bag, it can result in zonular dehiscence and dislocation of the IOLs. Based on the patient’s corrected distance visual acuity, fibrotic tissue appearance, and residual anterior chamber opening, previous researchers have clinically graded the ACCS (Table 2) (23). With the continuous advancement in research on ACCS, early intervention for high-risk patients has led to a reduction in the number of severe ACCS cases. Future studies should focus on determining the precise timing of capsular contraction, in order to better determine the appropriate follow-up schedule.

**Table 2:** Classification of anterior capsular contraction syndrome.

Grade^a^	Residual AC opening (mm)	Fibrotic tissue appearance	IOL status	Refractive changes	Visual symptoms
I	<4.0	Dense white fibrosis ring at capsulotomy edge	Haptic deformation	None to minimal	None to mild night glare, light sensitivity
II	<3.0	Fibrosis ring visible in pupil margin	IOL tilt <10° or IOL decentration <1.0 mm Zonule stress	SE change >0.5 D/internal astigmatism	Decrease in CDVA <2 lines
III	<2.0	Fibrosis ring in central visual zone (2.0 mm), possibly asymmetric	IOL tilt >10° or IOL decentration >1.0 mm IOL optic deformation	Internal astigmatism, HOAs	Decrease in CDVA >2 lines image distortion
IV	<1.0	IOL surface covered by dense white phimosis material	Loosening of capsular bag or ciliary body detachment	Refraction not measurable	Decrease in CDVA >3 lines

AC, anterior capsule; CDVA, corrected distance visual acuity; HOAs, higher order aberrations; IOL, intraocular lens; SE, spherical equivalent.

a From this table, 3 of 5 columns must be fulfilled to be graded I to IV.

## 4 Influencing factors for ACCS

### 4.1 Impact of CCC on ACCS

The direction of changes in the capsulorhexis area diameter is directly related to the size due to the forces generated during fibrosis contraction (14). An appropriately sized CCC maintains the opening area of the anterior capsule during postoperative capsular fibrosis, ensuring a stable position of the IOL within the capsular bag and preventing PCO (25, 26). Through the comparison of openings with different capsulorhexis diameters, Joo et al. (24) propose that a CCC larger than 5.5 mm exhibits an increasing trend in the subsequent changes of the opening area of the anterior capsule, while a CCC smaller than 5.0 mm results in a gradual reduction in the opening area of the anterior capsule. When the CCC is too small or off-center, postoperative wrinkling and contraction of the capsular bag can lead to a decrease in the opening area of the anterior capsule and even the occurrence of IOL decentration (24). However, the capsulorhexis area larger than 6.5 mm can damage the attachment point of the zonules, reducing the safety of the surgical procedure and causing zonular laxity. Therefore, when implanting an IOL with an optic diameter of 6.0 mm, the capsulorhexis area diameter should be in the range of 5.5 to 6.0 mm. Our team also recommends performing such diameter for highly myopic patients (27). In order to reduce the incidence of ACCS in patients at high risk of capsular contraction, it is essential to conduct further research to determine the optimal capsulorhexis size.

### 4.2 Impact of IOL characteristics on ACCS

#### 4.2.1 Optic of IOL

It has been observed that IOL optic materials have a significant impact on this condition among the controllable factors (28). The materials of optic can also be classified based on their chemical properties, such as silicone gel, hydrogel polymethyl-methacrylate (PMMA) and acrylate acid. Several previous studies have consistently reported that the extent of ACCS is greater after implantation of silicone or hydrogel IOLs compared to PMMA or acrylic IOLs (28, 29). However, currently, acrylic IOLs are the most commonly used in clinical practice, with PMMA IOLs being almost obsolete. Furthermore, in terms of the acrylic IOLs’ reaction to water, IOLs can also be categorized into hydrophilic and hydrophobic materials. Chen et al. (30) conducted a large-scale meta-analysis which showed that greater ACCS occurred in hydrophilic IOL groups compared to hydrophobic IOL groups at postoperative 1 month, 3 months, 6 months, and 1 year, which is consistent with most studies (31). The findings may be explained by the adhesion of optic materials to the capsule. During the early stages following implantation, the superior biocompatibility of hydrophobic IOLs enables them to securely adhere to the capsule, limiting space for LECs proliferation and ECM synthesis. Consequently, this reduces fibrosis and contraction of the anterior capsule (32). In order to improve the biocompatibility of optic, scientists have started to modify IOLs and have achieved promising results (33). In the future, it will be necessary to further compare the impact of different materials on ACCS and develop new types of IOL materials.

The optical edge of the IOL includes sharp-edged and round-edged. In recent years, extensive research on PCO has confirmed that square-edged designed IOLs can inhibit the growth of LECs from the peripheral capsular bag towards the visual axis, thereby preventing posterior capsule opacification (34). However, the impact of such IOLs on ACCS remains unclear. Recent research has revealed that an anterior edge design with a “peak-like” shape is more effective than a flat design in preventing the spread of LECs towards the edge of the anterior capsule opening. It helps to maintain the morphology of the anterior capsule opening during the early stages after surgery (35). Additionally, a few studies have indicated that certain factors, such as single-piece design or IOLs with thin optics, may increase the risk of capsule contraction (32, 36). However, research on the optic design is still far from sufficient, and further in-depth studies are needed to understand its impact on ACCS.

#### 4.2.2 Haptic of IOL

The haptic component of a IOL plays a crucial role in the interaction with the capsular bag. Regarding the materials commonly used for IOL haptics, including PMMA, polyvinylidene fluoride (PVDF), and acrylate, current studies have indicated that the material of IOL haptics does not have a significant impact on capsule contraction (6, 37). Some studies have compared an IOL with a PMMA haptic and a hydrophobic acrylic optic (Hoya YA60BBR; Hoya, Tokyo, Japan) to other commonly used IOLs. Although no statistically significant differences were found, it was observed that the ACCS of this type of IOL was less than others at 3 months postoperatively. Additional researches are required to explore the IOLs that effectively minimize ACCS.

In contrast to the optic, the haptic of the IOL has a greater mechanical influence on the capsulorrhexis area through direct interaction with the capsular bag (38). As the lens capsule is surrounded by the zonule system, an imbalance of forces can occur due to unmatched number, position, and shape of the haptics (35). For instance, plate haptic IOLs are known to exhibit more pronounced ACCS due to less capsule dilation caused by the centrifugal haptics (38). Moreover, the number and arrangement of haptics may interact with the capsule in a more complex manner. Studies have indicated that IOLs with four haptics can provide more precise fixation within the capsule, resulting in a larger contact area and more stable capsulorhexis margins (39). Choi et al. (38) suggested that to prevent ACCS, it is essential that the haptics uniformly support the zonule. For hinge-based accommodative IOLs, the presence of a hinge makes it more susceptible to the effects of ACCS, which can impact surgical outcomes (40). However, there is currently a lack of research on whether the presence of a hinge can trigger ACCS. Exploring effective haptic designs that minimize ACCS remains a critical area for additional research and study.

### 4.3 Impact of ocular and systemic diseases on ACCS

#### 4.3.1 High myopia

**Figure 1:**
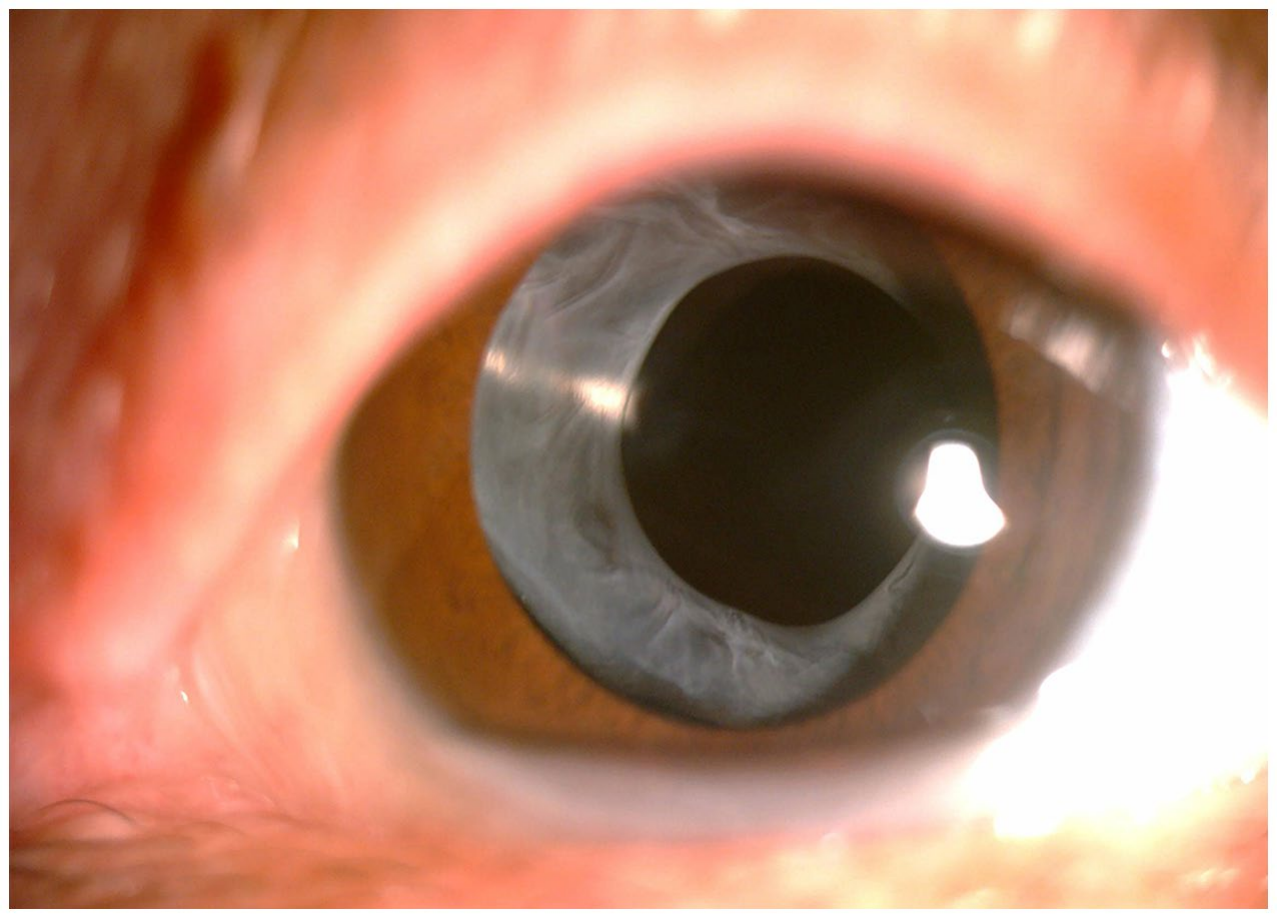
Anterior segment photograph of anterior capsular contraction syndrome in a high myopia patient.

High myopia, defined by a refractive error exceeding − 6.0 diopters (D) or an axial length (AL) greater than 26 mm, is more common in Asian populations (27). According to our research based on a large sample of over 4000 cataract patients, the incidence of ACCS in highly myopic cataract patients was reported to be 2.1%, which is significantly higher compared to the incidence rate of 0.15% to 0.86% observed in the general population with age-related cataracts (41). Tehrani et al. (42) proposed that the degree of capsular contraction is positively correlated with AL, based on their measurements of eye axis and postoperative capsular bag diameters in 58 patients within 6 months after surgery. This implies that as the AL increases, the degree of capsular contraction also increases. As the axial length of the eye increases, there can be elongation and weakening of the zonular processes. This can be attributed to the imbalance between the size of the lens and the size of the eyeball (15). Additionally, the heightened levels of growth factors and proinflammatory status in highly myopic eyes are believed to play a role in the proliferation of LECs, leading to the development of ACCS (43). Our team identified elevated levels of TGF-β2 in the aqueous humor and upregulated expression of TGF-βRII (the type II receptor for TGF-β2) in LECs in highly myopic cataract patients, particularly in those with dark nuclei. This finding suggests that the severity of ACCS may be related to the severity of cataracts (27). Both inflammation and zonular weakness have a combined impact on the development of ACCS in highly myopic patients. In more severe cases, as Jeon and Kim reported, the incidence of IOL dislocation in highly myopic patients was significantly greater compared to the general patient population (44). Therefore, in Chinese patients, high myopia is the most common risk factor for in-the-bag IOL dislocation (45). Hence, in clinical practice, for highly myopic patients, it is advisable to select IOLs with milder capsular contraction and implement appropriate intervention measures to prevent the occurrence of severe ACCS and even IOL dislocation (Figure 1).

#### 4.3.2 Retinitis pigmentosa

**Figure 2:**
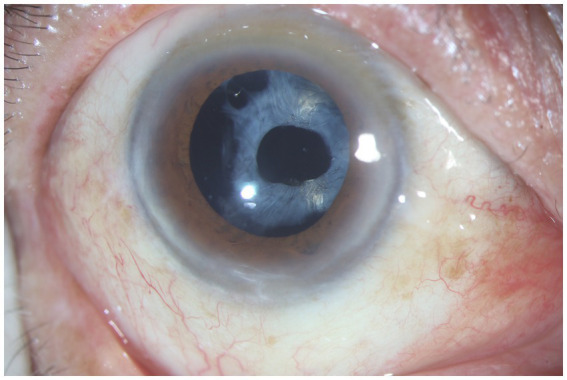
Anterior segment photograph of anterior capsular contraction syndrome in a retinitis pigmentosa patient.

The association between ACCS and RP was initially reported by Hayashi et al. (46). On the one hand, in patients with RP, zonular weakness has been documented and is associated with the imbalance between the outward zonular force and inward force caused by capsular fibrosis at the capsulorrhexis margin (47). The degeneration of zonule might be caused by a lipid peroxidative mechanism (48, 49). Researches have also demonstrated that degeneration of photoreceptors can lead to an upregulation of pro-inflammatory cytokines and chemokines, resulting in the development of a chronic inflammatory state in patients with RP (50). RP was also reported to be one of the predisposing factors of late spontaneous IOL-capsular bag complex dislocation (49, 51). On the other hand, recently, numerous studies have confirmed the role of inflammatory response and cytokines, such as platelet-derived growth factor AA (PDGF-AA), matrix metalloproteinase-2 (MMP-2), MMP3, MMP-7 and MMP-8, in the development of ACCS following cataract surgery in RP patients (52, 53). That may reveal the pathogenesis of ACCS in RP patients. Additionally, the liquefaction of the vitreous may lead to postoperative insufficient support of the capsular bag, which could potentially become one of the reasons for IOL dislocation (53). Further works are required to determine the conclusive evidence of the pathogenesis to identify targeted and effective therapeutics, and specific prevention to limit the occurrence of surgical complication (Figure 2).

#### 4.3.3 Uveitis

**Figure 3:**
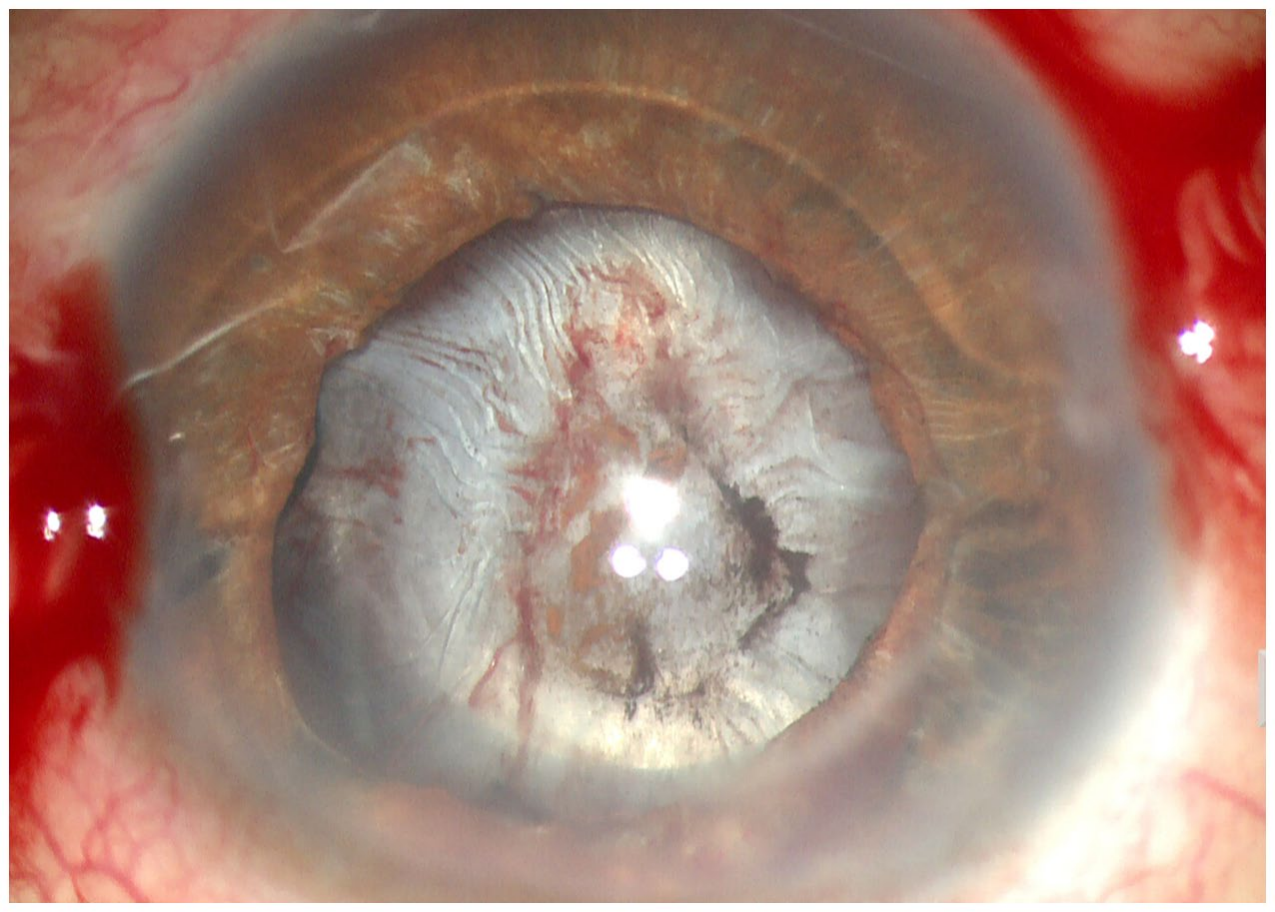
Anterior segment photograph of anterior capsular contraction syndrome in a chronic iridocyclitis patient.

**Figure 4:**
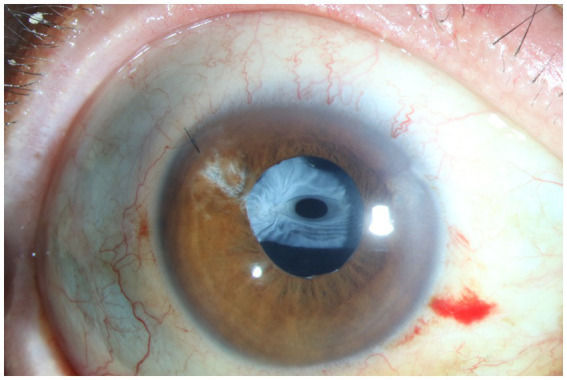
Anterior segment photograph of anterior capsular contraction syndrome in a traumatic sympathetic ophthalmia patient.

Uveitis is a prevalent ocular condition characterized by inflammatory processes that cause structural and functional damage to both the anterior and posterior segments of the eye (54). In the past, cataract surgery was not recommended for patients with intraocular inflammation due to the higher risk of complications after the procedure (55). In uveitis cases, it is possible for inflammation to extend to the pars plicata, leading to zonular instability. The inflammation of the ciliary body can exacerbate the destabilization of the zonules at their insertion sites. This is related to the adherence of white blood cells or fibrin on the surface of the ciliary body (56). Moreover, patients with uveitis not only experience inflammation associated with the condition itself but also in relation to the cataract surgery procedure. The presence of inflammatory factors stimulates LECs to secrete an increased amount of growth factors, which promotes fibrotic growth of the capsular bag. This ultimately increases the risk of early occurrence of ACCS (54). In the study conducted by Chen et al. (57), it was found that the active inflammation tends to subside by 3 months, while the restoration of blood-aqueous barrier function may require a longer period of time. In order to minimize the occurrence of postoperative ACCS, it is of utmost importance to adequately control inflammation prior to surgery, ensure meticulous surgical technique, and enhance anti-inflammatory measures during the postoperative period (58, 59). Nevertheless, uveitis is a broad classification, and further research is needed to understand the impact of different types of uveitis on ACCS (Figures 3, 4).

#### 4.3.4 PEX syndrome

PEX is a disorder of the ECM that is associated with aging and can result in various ocular complications (60). The prevalence of PEX shows significant variation based on geographic location and ethnicity. Studies examining specific populations have revealed a higher occurrence (ranging from 25% to 30%) in certain ethnic groups, including northern Scandinavians, Saudi Arabians, and Navajo Indians (61, 62). Previous studies have indicated that the accumulation of PEX material causes mechanical weakening of the zonule and disrupts their attachment to the ciliary epithelial basement membrane, both at their origins and insertions. Moreover, PEX eyes demonstrate heightened elastinolysis, resulting in enzymatic degradation of the zonules and increased fragility (63). Ruotsalainen and Tarkkanen (64) conducted a study and concluded that the elevated rate of complications observed in eyes with PEX could be attributed to the presence of relatively fragile zonules commonly found in PEX cases. Furthermore, it has been reported that PEX can compromise the integrity of the blood-aqueous barrier (65, 66), leading to the increased release of inflammatory cytokines and ECM material into the anterior chamber. This can further contribute to the occurrence of ACCS. Additionally, PEX is the most common underlying condition for spontaneous in-the-bag IOL dislocation (19). It is currently believed that implanting a CTR or postoperative use of nonsteroidal anti-inflammatory drugs (NSAIDs) can be effective in reducing postoperative ACCS in patients with PEX (62, 67, 68). Currently, many substances have been found to be possibly associated with PEX and ACCS. Identifying and potentially distinguishing factors involved in the complex pathophysiological steps corresponding to LEC stress response remains a priority, which may help prevent the occurrence of these two complications.

#### 4.3.5 Diabetes

Takamura et al. (69) found that diabetes mellitus serves as a systemic risk factor for capsule contraction, especially in the presence of diabetic retinopathy (DR), when comparing the ACO area with nonproliferative DR, those without DR, and those without DM. Further, the intensity of aqueous flare in diabetic patients 1 week after surgery was significantly correlated with the severity of ACCS postoperatively. On the other hand, researchers have demonstrated through immunohistochemical analysis that LECs in diabetic eyes exhibit higher levels of IL-1 and TGF-β activities compared to normal eyes. Diabetic eyes show a stronger proliferation of LECs compared to normal eyes (9). The glycemic control could potentially impact the severity of postoperative ACCS. However, despite a case report (70), there is currently a lack of research investigating the relationship between patients’ blood glucose or glycated hemoglobin levels and the severity of ACCS.

As for intervention for ACCS in diabetic patients, preventing anterior inflammation may play a critical role in inhibiting the development of postoperative ACCS. NSAIDs have been demonstrated to inhibit the synthesis of prostaglandins, suppressing anterior inflammation (69). Recently, Baldysiak-Figiel et al. (71) have discovered that octreotide exhibits the capacity to decrease proliferative responses of LECs and effectively inhibits cell proliferation induced by growth factors. This suggests that octreotide shows potential as a medication for preventing growth factor-related proliferative complications, including PCO and ACCS, in diabetic patients undergoing cataract surgery.

#### 4.3.6 Dystrophia myotonica

Dystrophia myotonica, an autosomal dominant disease, is caused by a mutation in the dystrophia myotonica protein kinase gene. It has been found to have a higher prevalence in European regions compared to non-European regions, and it was observed to be rare in East Asia and sub-Saharan Africa (72). Some researchers proposed that in dystrophia myotonica, the atrophy of the ciliary body may lead to an imbalance of forces, specifically an unopposed centripetal force at the margin of the capsulorhexis (73). It is possible that LECs in individuals with dystrophia myotonica have a higher tendency to undergo an exaggerated fibroblastic, proliferative response after cataract surgery, leading to increased contractility following metaplasia to myofibroblasts. Genetic examinations of LECs in dystrophia myotonica patients have revealed the presence of the dystrophia myotonica-protein kinase (DMPK) gene mutation in these cells (74). However, there is only a few research in this area and more studies are needed to gain further insights.

#### 4.3.7 Other disease factors

In clinical practice, in addition to the aforementioned conditions, other diseases have been reported to be associated with ACCS, such as chronic angle-closure glaucoma (75, 76), Marfan syndrome (77) and those following full-thickness penetrating keratoplasty (78). These may also be associated with weakened zonules or inflammatory responses. However, currently, there is a lack of research revealing the correlation between these diseases and ACCS. It is anticipated that more studies in these fields will be conducted in the future to shed light on these associations.

### 4.4 Other influencing factors

Many studies have indicated that advanced age (≥80 years old) is a risk factor for ACCS after cataract surgery, possibly due to the increased fragility of zonules in elderly individuals (4, 20). This suggests that the older the patient, the more important it is to implement appropriate interventions. Recent study has indicated that in congenital cataract patients who undergo surgery at a younger age, there is a significantly increased level of proinflammatory cytokines in the aqueous humor postoperatively. However, there is no statistically significant correlation between these cytokine levels and postoperative capsular contraction (10). Further research could explore the relationship between age and the incidence of ACCS, but it is essential to consider the differences in the etiology of cataract patients across different age groups.

## 5 Interventions for ACCS

### 5.1 Intraoperative intervention methods

#### 5.1.1 Anterior capsule polishing

Reducing the number of LECs can be considered as one of the intervention methods for postoperative ACCS. Currently, several approaches are being employed in clinical practice to clear LECs, including the use of antimetabolic drug mitomycin (79), drug-loaded delivery systems (80), and physical treatments (81) such as heating or cryotherapy. However, each of these approaches has its limitations.

In contrast, anterior capsule polishing is considered most widely used method (82–84). The ultrasound irrigation and aspiration tip has been identified as the most effective instrument for mechanical polishing (85). Numerous studies have demonstrated that the aspiration of LECs from the anterior lens capsule is a beneficial approach in preserving the size of a capsulorhexis, subsequently serving as a preventive measure against ACCS (83, 86, 87). The study by Zhao et al. (88) compared a group of cataract patients in which anterior capsule polishing was performed in one eye and not in the other. They successfully demonstrated that this approach reduces the severity of anterior capsule contraction and enhances the stability of IOLs in highly myopic patients. However, the efficacy of anterior capsule polishing during phacoemulsification surgery continues to be a topic of debate and controversy (89). Also, as the cataract becomes more mature, the zonule become increasingly delicate (90), eventually leading to the IOL dislocation, a condition in which the aspiration of anterior epithelial cells can become remarkably challenging or even unfeasible. Thus, for patients at high risk of ACCS, a thorough examination of the zonular condition is essential before considering anterior capsule polishing. Combining anterior capsule polishing with CTR implantation may be a safer approach.

#### 5.1.2 Implantation of CTR

The implantation of CTRs provides centrifugal force to redistribute forces between existing zonules, preventing force concentration and protecting fragile areas. This effectively prevents significant capsular bag shrinkage in eyes with or without zonular weakness. This preservation plays a crucial role in preventing substantial decentration and tilt of the IOL, as well as the occurrence of severe ACCS. This helps to maintain the effective position and stability of the IOL, improve higher-order aberrations, and enhance visual quality (91). Therefore, we recommend the intraoperative implantation of CTR for cataract patients who have risk factors, as a preventive measure against postoperative ACCS (30, 92). For instance, a retrospective study conducted on patients undergoing cataract surgery with RP revealed that the incidence of ACCS was lower in those who received CTR implantation compared to those without CTR (93). In a 3 months follow-up study of 20 highly myopic patients with axial length exceeding 28 mm, Yang et al. (94) found that those who received CTR implantation had lager ACO area and less pronounced IOL tilt compared to those without CTR implantation. However, even with the implantation of a CTR, complete prevention of IOL dislocation may not be guaranteed (95). Interestingly, although there is limited similar research, Vanags et al. (96) compared the effect of a basic (11 or 12 mm) or Cionni (12 mm) CTR on ACCS. They observed differences in the effects at 1 month after the surgery, with the larger diameter CTR demonstrating better intervention outcomes. Looking ahead, further research into CTRs is expected to shed light on their optimal use and effectiveness in preventing ACCS and IOL dislocation. Moreover, in recent years, several modified CTR designs have emerged, awaiting further investigation and study.

#### 5.1.3 IOL modification

The biocompatibility of IOL is a crucial aspect in minimizing postoperative inflammation. In the past, IOL modification, especially the utilization of heparin-surface-modified (HSM) IOLs, has been widely employed for several years to enhance their biocompatibility. The application of heparin surface modification technology was initially introduced to PMMA lenses in the early 1990s, and it has demonstrated notable benefits in reducing inflammation (97). It can effectively mitigate the breakdown of the blood-aqueous barrier and diminish the foreign body response and imparts a negative charge to the surface of IOLs, thereby reducing the associated complications (98). In studies conducted by Krall et al., the inflammation levels of HSM hydrophobic acrylic IOLs were compared to un-coated acrylic IOLs. The findings revealed that in the early postoperative stage, the HSM IOL group exhibited lower inflammation levels compared to the un-coated IOL group. However, Maedel et al. (99) found that the heparin surface modification had no impact on postoperative ACCS when implanting hydrophobic acrylic IOLs. Furthermore, Tan et al. (100) conducted a new synthesis of a hydrophilic copolymer, and the resulting modified IOL demonstrated a significant reduction in postoperative inflammation and ACO. We can anticipate the development of more modified materials that can be used to prevent ACCS.

### 5.2 Postoperative intervention methods

#### 5.2.1 Anti-inflammatory medications

Inflammatory response is a significant factor that influences capsular contraction. Therefore, it is crucial to administer anti-inflammatory treatment before and after the surgery to high-risk patients with significant inflammatory reactions (101). Researchers have found that both NSAIDs and corticosteroids are effective in preventing ACCS in rabbit eyes (102). On the other hand, both corticosteroids and NSAIDs are associated with noticeable side effects when used long-term, which necessitates caution, particularly in patients such as PEX, RP, and uveitis (103). However, there is currently limited research on the use of anti-inflammatory drugs for preventing ACCS. Further studies are needed to explore this area in depth.

#### 5.2.2 Laser anterior capsulotomy

**Figure 5:**
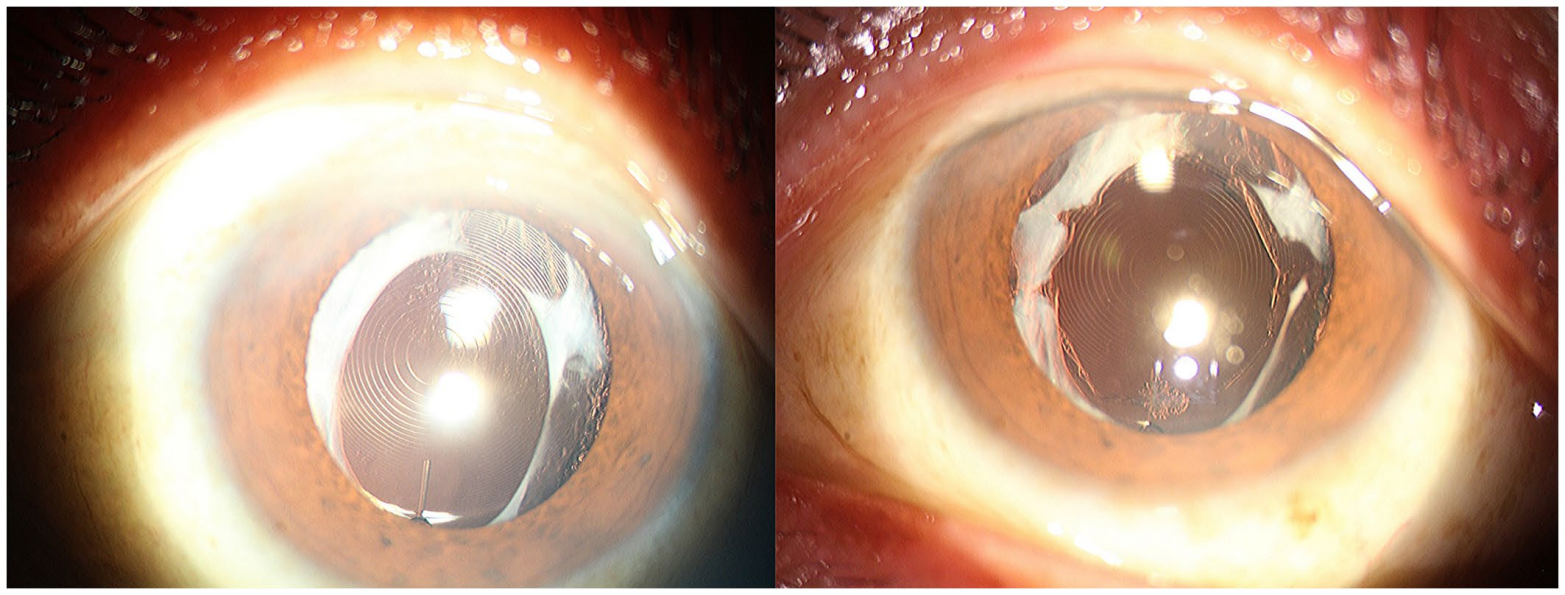
Anterior segment photographs in a patient with capsular contraction syndrome before (left) and after (right) Neodymium:YAG laser anterior capsulotomy.

To prevent the occurrence of ACCS, Davison first proposed performing Nd:YAG laser anterior capsulotomy 2–3 weeks after cataract surgery (4). For patients with mild cases of ACCS, it is a simple non-surgical treatment option. Nd:YAG laser primarily works through the ionization effect, generating plasma within the target tissue. It utilizes the shockwave produced by the resulting explosion to disrupt and disintegrate the tissue. This makes it an ideal non-surgical choice for treating ACCS (104). Hayashi et al. (105) analyzed the anterior capsulotomy area before and after Nd:YAG laser capsulotomy in 32 eyes, and found that contrast sensitivity at most visual angles significantly improved following the procedure. The way of laser incision needs to be adjusted based on the degree of wrinkling in the anterior capsule. In the early stages of capsular contraction, before any visual impairment occurs, incisions can be made near the area of contraction. When the size of the anterior opening is smaller than the size of the pupil and accompanied by fibrotic proliferation of the anterior capsule, a radial Nd:YAG laser capsulotomy should be performed in all four quadrants. Interestingly, Hayashi et al. (106) compared the effects of different numbers of incisions and found that three relaxing incisions made in the anterior capsule decrease anterior capsule contraction, whereas two incisions do not. Further, it is reported that more than four incisions without extending beyond the IOL optic edge are needed to prevent free-floating remnants from dropping into the anterior chamber, impacting the sight during reading (107). Regarding the capsulotomy method, radial incision is currently the most widely used. Elmohamady et al. (108) found that circular Nd:YAG anterior capsulotomy is considered more effective and safe than radial capsulotomy in a 1 year follow-up study. However, it is important to note that capsular contraction or Nd:YAG laser anterior capsulotomy carries the risk of zonular breakage or weakening. Furthermore, anterior capsulotomy alone cannot halt the progression of intrinsic zonular weakness (109, 110). Complications such as hyphema, inflammation, and elevated intraocular pressure can occur as secondary effects of Nd:YAG laser anterior capsulotomy (111). With the advancement of technology, the utilization of femtosecond laser technology in cataract surgery has allowed for a less invasive and safer approach, minimizing injury to the zonules. This technique has been widely adopted to effectively and safely enlarge the capsulorhexis (112). Further research is needed to investigate whether a femtosecond laser can offer advantages over existing treatment methods in intervening ACCS (Figure 5).

#### 5.2.3 Surgical methods for intervention in ACCS

**Figure 6:**
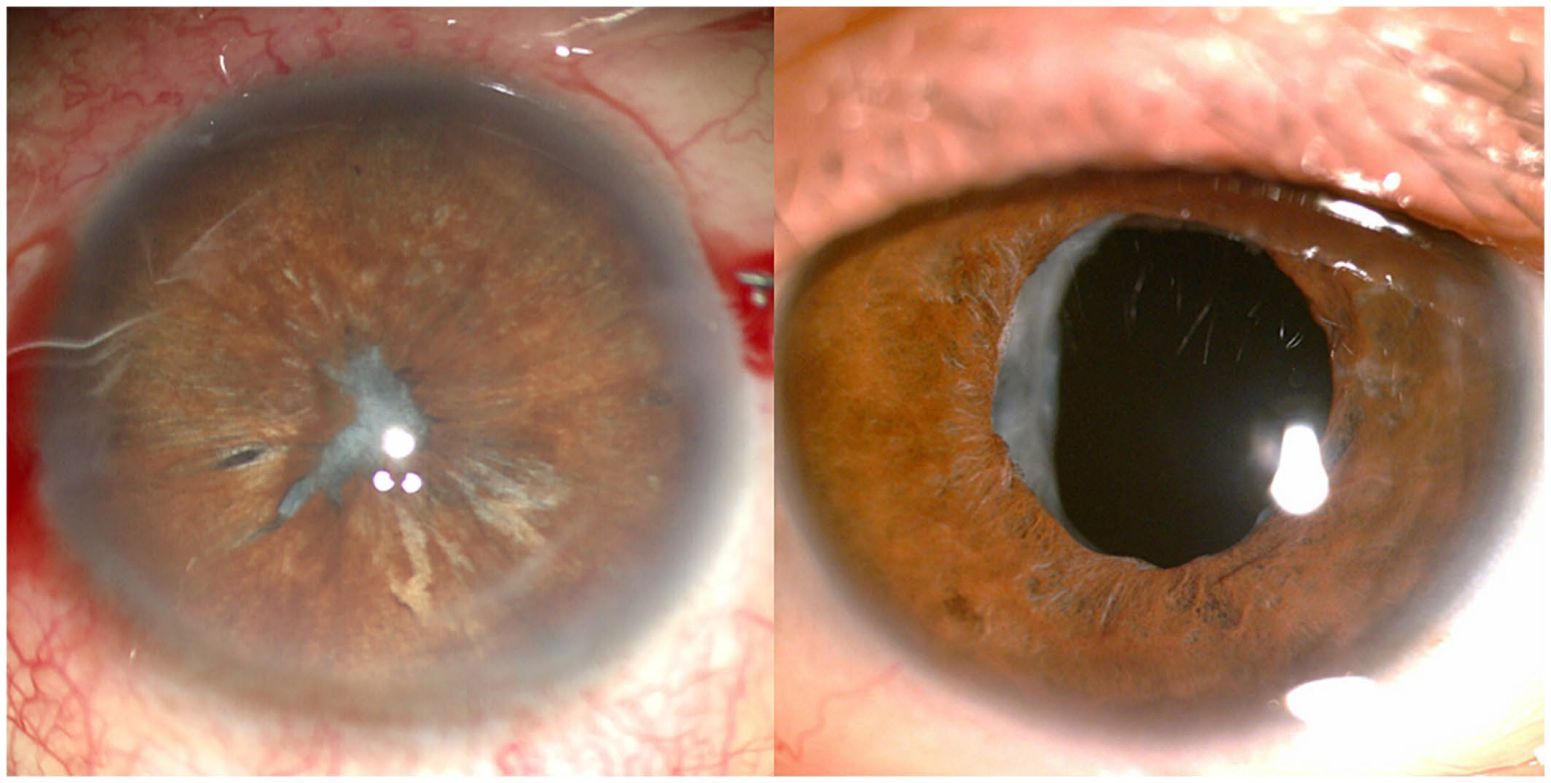
Anterior segment photographs in a patient with capsular contraction syndrome before (left) and after (right) capsular bag relaxation surgery.

For patients with severe ACCS, the presence of densely hyperplastic fibers that cannot be disrupted by laser treatment poses a challenge. Moreover, the large fragments of fibrous membrane that result from the disruption of hyperplastic fibers may not be absorbed spontaneously. In these cases capsular bag relaxation surgery (CBRS) emerges as a suitable alternative (113). The CBRS procedure in this study involved two techniques: actinoid relaxing incision and secondary CCC, the latter is mainly used for cases where the anterior capsular opening is approaching closure. The traditional method mainly use capsulorhexis forceps, whereas Yeh et al. (114) proposed using a vitrector handpiece to achieve a more circular opening, albeit at the cost of increased time consumption. However, it is worth noting that this method may have drawbacks when dealing with poor zonular function, as it can potentially cause zonule damage and result in IOL deviation (40). Additionally, for patients with severe conditions such as IOL dislocation, anterior capsule release may be not effective. In such cases, procedures such as IOL suspension or ciliary sulcus suture fixation may be necessary to restore good visual acuity for the patient (115). Importantly, it should be clear that thorough preoperative assessment and proactive postoperative prevention are essential. These are far more important than surgical treatment (Figure 6).

## 6 Conclusion

In conclusion, ACCS remains a challenging complication in cataract surgery, affecting both the visual outcomes and patient satisfaction. Important factors influencing capsular contraction include surgical techniques, choice of IOL, intraocular inflammation and various diseases. Interventions include avoiding excessive manipulation of intraocular tissues, utilizing IOLs with good biocompatibility, managing intraocular inflammation appropriately, and regular follow-up with prompt management of early capsular contraction. These interventions are vital in reducing surgical complications and improving surgical outcomes. Future studies should focus on long-term follow-up and comparative analyses to determine the most successful interventions for preventing and managing ACCS. Ultimately, with continued research and advancements, we can strive towards minimizing the occurrence of this syndrome and optimizing visual results for cataract surgery patients.

## Author contributions

XL: Conceptualization, Investigation, Writing – original draft, Writing – review & editing. DM: Investigation, Writing – review & editing. JY: Conceptualization, Supervision, Writing – review & editing.
